# Graphene Oxide (GO)/Polyamide Thin-Film Nanocomposite Membranes for Enhanced Pervaporation Desalination: Role of GO in Microstructural Modification and Water Transport

**DOI:** 10.3390/membranes16100337

**Published:** 2026-10-11

**Authors:** Yi Chen, Yuxuan Li, Yaoli Guo, Xiaolong Xie, Pengwei Huang, Weini Chen, Shengchao Zhao

**Affiliations:** 1 Department of Chemical Engineering, Jieyang Polytechnic, Jieyang 522000, China; 2 MayAir Technology (China) Co., Ltd., Nanjing 210000, China; 3 State Key Laboratory of Radio Frequency Heterogeneous Integration, Shenzhen University, Shenzhen 518060, China; 4 Institute for Advanced Study, Shenzhen University, Shenzhen 518060, China; 5 College of Civil and Transportation Engineering, Shenzhen University, Shenzhen 518060, China

**Keywords:** graphene oxide, polyamide, thin-film nanocomposite membrane, pervaporation desalination, molecular dynamics

## Abstract

Pervaporation (PV) desalination offers a viable route for high-salinity wastewater treatment, yet its broader implementation is constrained by limited membrane permeability. In this study, graphene oxide (GO)/polyamide (PA) thin-film nanocomposite (TFN) membranes were prepared through interfacial polymerization, wherein GO nanosheets were pre-dispersed in the aqueous m-phenylenediamine phase at different loadings. The prepared membranes underwent comprehensive characterization to evaluate the effects of GO incorporation on surface compositions and properties. Complementary molecular dynamics simulations were employed to explore the molecular-scale water diffusion within the membrane matrix. The results demonstrated that GO effectively enhanced the surface hydrophilicity, reduced the selective layer thickness, rendered the water–membrane binding energy more negative, and increased the fractional free volume, collectively boosting the pervaporation flux. The optimal membrane delivered a flux of 22.38 L m^−2^ h^−1^, surpassing the pristine PA membrane by 31%, while maintaining a salt rejection of 99.9% at 70 °C using a 3.5 wt% NaCl feed solution. It also exhibited stable performance at higher salinities, maintaining a flux of 14.4 L m^−2^ h^−1^ and rejection above 99.9% at 10 wt% NaCl. This work provides a straightforward protocol for PA-based TFN membranes with enhanced performance in PV desalination, offering molecular-level insights into the role of GO in modifying membrane microstructure and transport properties.

## 1. Introduction

Desalination via membrane separation is now established as a key strategy for addressing global water shortages, owing to its low energy consumption, operational reliability, and ease of scale-up [1,2,3]. Among various membrane processes, pervaporation (PV) is recognized as a particularly promising approach for treating high-salinity wastewater and industrial brine [4,5,6]. In contrast to pressure-driven reverse osmosis, pervaporation relies on a vapor-pressure driving force. In PV desalination, water selectively permeates through the dense membrane as vapour, while non-volatile solutes such as salt ions are rejected due to their inability to vapourise and transport through the polymer matrix, thereby achieving high salt rejection. This mechanism renders the separation efficiency of PV largely independent of feed salinity [7,8,9]. Moreover, compared with membrane distillation, PV also offers superior resistance to wetting and fouling, as well as lower pretreatment requirements [10,11]. These intrinsic characteristics make PV a compelling option for hypersaline wastewater treatment and zero-discharge scenarios, which are increasingly required in modern industrial water management [12,13,14].

However, a persistent limitation of PV membranes is their modest water permeance, which constrains process economics at scale [15,16,17]. While dense membrane structures have been conventionally employed in PV desalination, asymmetric membranes featuring an ultrathin, dense selective layer on a porous substrate have emerged as an effective strategy for enhancing permeation without compromising salt rejection [10,18]. In this context, thin-film composite (TFC) membranes synthesised via interfacial polymerization (IP) have been increasingly adopted for PV desalination, owing to their high permeability and straightforward fabrication [19,20]. The dense, highly cross-linked polyamide (PA) active layer endows TFC membranes with greater swelling resistance than PVA-based membranes [18]. Nevertheless, the intrinsically low hydrophilicity and dense microstructure of PA limit its water flux in PV desalination, necessitating further structural modification.

Introducing nanomaterials to regulate molecular diffusion pathways and improve hydrophilicity in thin-film nanocomposite (TFN) membranes can effectively enhance the separation efficiency in pervaporation desalination [19,21,22,23,24]. Graphene oxide (GO) nanosheets are increasingly favored as TFN fillers due to their two-dimensional structure, oxygen-rich surface chemistry, and excellent hydrophilicity [25,26,27,28]. When integrated into PA TFN membranes via embedding, interlayer deposition, or surface modification, GO can enhance surface hydrophilicity, improve interfacial properties, and enhance water permeability without compromising salt rejection [29,30,31,32,33]. Zhao et al. constructed thin PA layers with a stripe-like structure on a GO-coated polyacrylonitrile substrate, achieving a water flux of 26.7 L m^−2^ h^−1^, while maintaining 99.99% salt rejection with a 3.5 wt% NaCl solution at 70 °C [34]. Halakoo et al. employed a chlorination–LbL hybrid strategy to modify PA membranes, in which electrostatic self-assembly of cationic PEI and anionic GO was performed on chlorinated PA supports. The optimized membrane exhibited a water flux of 8.4 kg m^−2^ h^−1^, double that of the pristine membrane, and >99.9% salt rejection when treating 20 wt% NaCl at 65 °C [35]. In our previous work, we fabricated a rod-coated PA membrane with controllable post-modification by GO. The modified membrane exhibited 99.99% salt rejection and a water flux of 22.6 L m^−2^ h^−1^ with a 3.5% NaCl feed at 50 °C [36]. While these experimental studies have established the effectiveness of GO incorporation in enhancing PV desalination performance, the molecular-level understanding of GO–PA interactions remains limited [37]. Specifically, how GO modulates the free volume distribution and water–polymer interactions that underpin the solution-diffusion process remains to be elucidated. Molecular dynamics (MD) simulations offer a powerful means of quantifying these structural descriptors, yielding mechanistic insights that are difficult to obtain through experiments alone [38,39].

In this study, we presented a facile one-step approach for fabricating high-performance GO/PA TFN membranes. This method directly incorporates varying concentrations of GO nanosheets (0.005–0.08 wt%) into the m-phenylenediamine (MPD) aqueous solution prior to interfacial polymerization, offering a more straightforward and scalable fabrication route compared to multi-step post-modification or interlayer coating strategies. The effects of GO incorporation on membrane surface chemistry, morphology, hydrophilicity, and surface charge were systematically characterized and analyzed. To unravel the underlying molecular mechanisms contributing to the enhanced pervaporation desalination performance, we employed MD simulations. Specifically, by calculating the water–membrane interaction energy and fractional free volume (FFV), our MD approach allowed us to decouple and elucidate the individual contributions of water solubility and diffusivity within the solution-diffusion process. This combined experimental and computational approach enables us to elucidate the role of GO in modifying membrane microstructure and water transport behaviour, providing molecular-level insights into the origin of the enhanced PV desalination performance of GO/PA TFN membranes.

## 2. Materials and Methods

### 2.1. Materials

Trimesoyl chloride (TMC, >98.0%) and m-phenylenediamine (MPD, >98.0%) were purchased from TCI (Tokyo, Japan). NaCl (≥99.5%) and n-hexane (≥97.0%) were supplied by Sinopharm Chemical Reagent Co., Ltd. (Shanghai, China). The polysulfone ultrafiltration substrate was obtained from Wave Cyber (Shanghai) Co., Ltd. (Shanghai, China). An aqueous dispersion of graphene oxide (5 mg mL^−1^) was provided by Suzhou Tanfeng Technology Co., Ltd. (Suzhou, China). All aqueous solutions were prepared using deionized water with a conductivity of less than 2 μS cm^−1^.

### 2.2. Membrane Fabrication

Polyamide selective layers were deposited on a polysulfone (PSf) support through IP reaction between aqueous MPD and organic TMC, with monomer concentrations and reaction conditions adopted from a previous optimization study [40]. Before use, PSf substrates were conditioned in DI water for 2 h to leach out the humectant and maintain hydration. After being fixed onto a polymethyl methacrylate frame with the active side upward, its surface was air-dried until no residual water was visible. Afterwards, an aqueous MPD solution (2 wt%) was introduced to saturate the substrate for 3 min. Excess solution was drained, and after brief surface drying, the activated substrate was exposed to a 0.1 wt% TMC solution for 1 min to initiate polyamide formation. Unreacted species were subsequently removed by washing with fresh n-hexane. To ensure sufficient crosslinking, the membranes were placed in an oven at 60 °C for 2 min. For the GO/PA TFN membranes, the same protocol was adopted, except that GO nanosheets at concentrations of 0.005–0.08 wt% were pre-dispersed in the MPD aqueous solutions. Before use, the GO/MPD dispersion was ultrasonicated for 30 min at room temperature to ensure uniform dispersion. Table 1 summarizes the GO concentrations and corresponding membrane labels. Figure S1 illustrates the morphological characterization of the commercial GO.

### 2.3. Membrane Characterization

Surface chemistry of the membranes was probed using Attenuated Total Reflectance Fourier Transform Infrared (ATR-FTIR) spectroscopy (Vertex 70, Bruker Optik, Ettlingen, Germany), X-ray Photoelectron Spectroscopy (XPS; Nexsa, Thermo Fisher, Waltham, MA, USA), and Raman Spectroscopy (Alpha 300 R, WITec, Ulm, Germany). Raman tests were conducted using a 532 nm laser as the excitation source. For XPS analysis, monochromatic Kα X-ray radiation (h_ν_ = 1486.6 eV) was used as the excitation source, with a working voltage of 15 kV and a filament current of 15 mA. The surface morphology and roughness of the membranes were examined by Atomic Force Microscopy (AFM, Innova, Bruker Nano, Santa Barbara, CA, USA) in tapping mode. Scanning Electron Microscopy (SEM; Quanta 650 FEG, FEI, Hillsboro, OR, USA) was used to examine the surface and cross-sectional morphology after gold deposition. A Contact Angle Analyzer (DSA25, Krüss, Hamburg, Germany) was used to assess surface hydrophilicity using the sessile drop method. Zeta potential measurements were performed using an Electrokinetic Analyzer (SurPASS 3, Anton Paar, Graz, Austria) to probe the surface charge.

### 2.4. Membrane Performance Test

**Figure 1:**
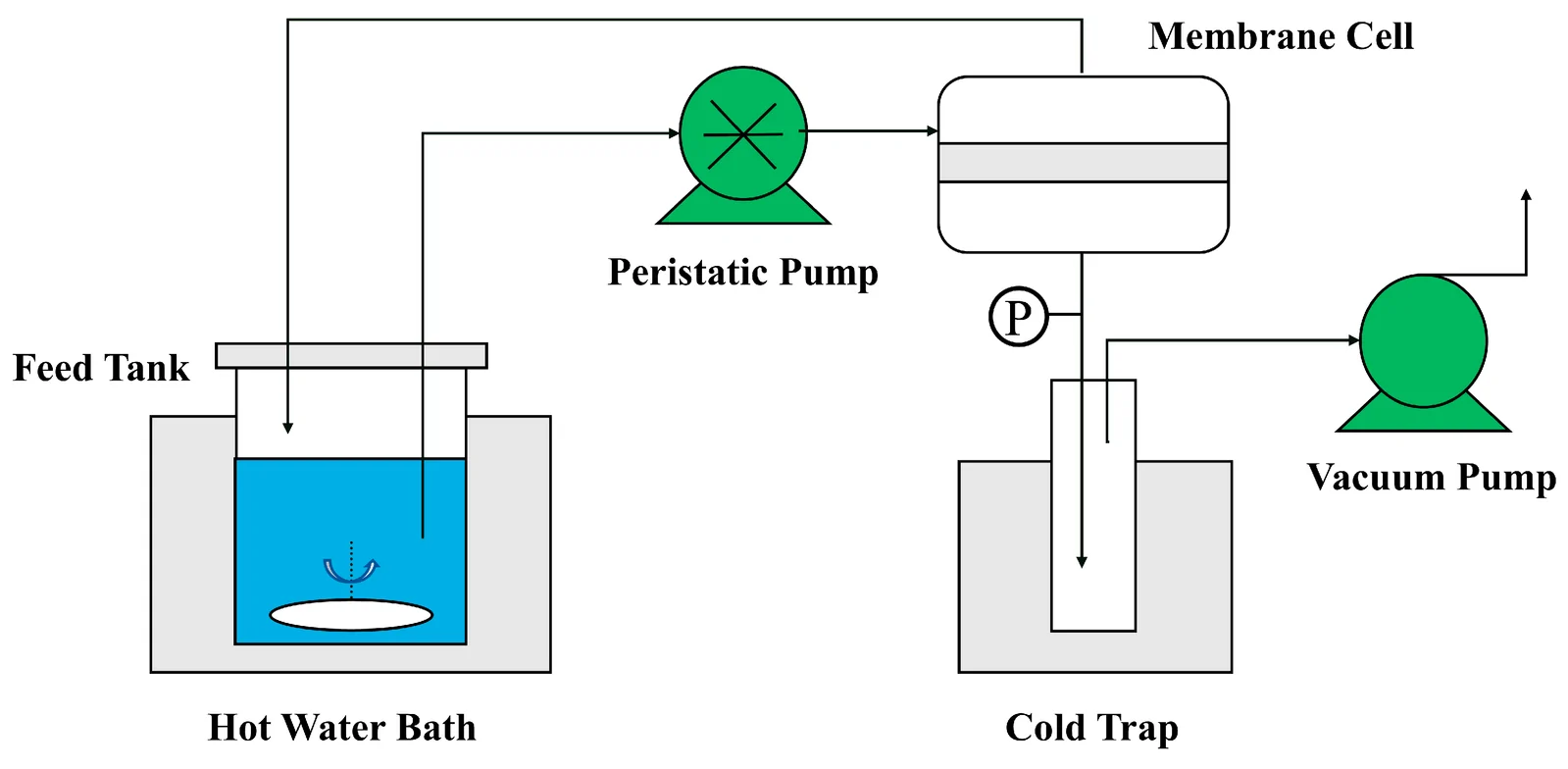
Schematic diagram of the pervaporation desalination test setup.

A homemade pervaporation setup (Figure 1) was used to test membrane performance, with an effective permeation area of 17.5 cm^2^. NaCl solutions of varying concentrations (typically 3.5 wt%) were used as the feed, and the feed temperature (typically 70 °C) was maintained using a hot-water bath. Feed flow through the membrane cell was set to 600 mL min^−1^ using a peristaltic pump (BT300L, Baoding Lead Fluid Technology Co., Ltd., Baoding, China). The permeate compartment was evacuated to ~1.5 kPa, and the vapour was liquefied in a cold trap held at ~5 °C by recirculating water. A VuRP8 vacuum pump (Shanghai Titan Technology Co., Ltd., Shanghai, China) was used to maintain the vacuum on the permeate side. After a 0.5 h stabilization period, the permeate mass was recorded. The water flux (J_w_, L m^−2^ h^−1^) was derived from:

(1) $$J_{w}=\frac{∆V}{A\cdot t}$$

where ΔV denotes the permeate volume (L). A (0.00175 m^2^) is the effective area of the permeation cell. And t is the collection time (h). The salt rejection (R, %) was computed as follows:

(2) $$R=\left(1-\frac{C_{p}}{C_{f}}\right)\times 100\%$$

where C_p_ and C_f_ are the salt concentrations of the permeate and feed solutions, respectively, which were determined by conductivity measurements (μS cm^−1^). The conductivity was measured using a conductivity meter (FE38, Mettler Toledo, Greifensee, Switzerland) with a detection limit of 0.01 μS cm^−1^ at room temperature. All membrane performance tests were conducted in triplicate using independently fabricated membrane samples.

### 2.5. Simulated Calculations

The calculations of the fractional free volume and water–membrane interaction energy of both PA TFC and GO/PA TFN membranes were performed using Material Studio 8.0 (BIOVIA, Dassault Systèmes, San Diego, CA, USA)with the COMPASS II force field. The PA network was constructed from 110 TMC and 165 MPD molecules (TMC:MPD = 1:1.5) using a cross-linking script developed by Jason DeJoannis et al. The cross-linking degree between fixed amino and acyl chloride groups was set at 70%, which is close to the experimental value determined by XPS analysis. The cross-linked amorphous cells subsequently underwent kinetic relaxation following a previously described protocol [41]. For the GO/PA TFN membrane model, two graphene oxide sheets (molecular weight = 522.424 g mol^−1^) were embedded into the PA amorphous cell, and the same cross-linking and relaxation protocol was applied to generate the nanocomposite network. This model should be interpreted as a qualitative probe of GO–PA interfacial interactions rather than a quantitative replica of any specific experimental membrane. Detailed simulation parameters and GO model specifications are provided in Section S2 of the Supporting Information (Figure S2).

The FFV was calculated using the Connolly surface method with a spherical probe of 2.8 Å in diameter, corresponding to the kinetic diameter of a water molecule. For interaction energy evaluation, a three-layer model (Figure S3) was assembled comprising a membrane layer (PA or GO/PA), a water layer (530 H_2_O molecules), and a vacuum slab (80 Å). After 150 ps NVT equilibration, the interaction energy between water molecules and membranes was determined using the script developed by Stephen Todd. More details are provided in Section S3 of the Supporting Information.

## 3. Results and Discussion

### 3.1. Surface Chemical Composition and Properties

**Figure 2:**
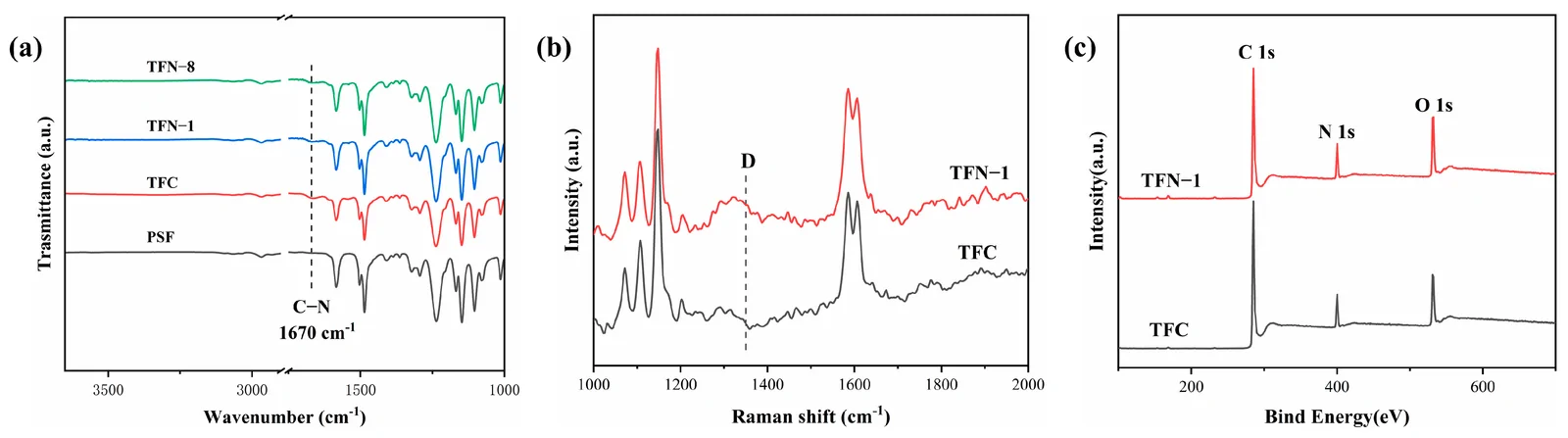
(**a**) IR spectra of the membranes; (**b**) Raman spectra of the TFC and TFN−1 membranes; (**c**) XPS survey spectra of the TFC and TFN−1 membranes.

The IR spectra of both TFC and TFN membranes showed a C–N stretch at 1670 cm^−1^, which was absent in the bare PSf support, indicating successful formation of the cross-linked aromatic PA layer (Figure 2a). However, due to low GO loading and the physical coverage by the rapidly formed PA layer, no obvious GO-specific peaks were detected in the TFN membrane IR spectra. The Raman spectrum of the TFN membrane exhibited slightly higher intensity and a broad, shoulder-like feature in the D-band region (~1350 cm ^1^) compared to that of the TFC membrane (Figure 2b). This subtle enhancement can be attributed to the superposition of the D-band signal from the embedded GO nanosheets. XPS analysis further confirmed a notable increase in surface oxygen content after GO incorporation. The TFN-1 membrane exhibited 16.16% oxygen versus 13.12% for the pristine TFC membrane. This increase is attributed to GO nanosheets and GO-induced changes in the PA layer’s chemical structure and cross-linking, which can alter interfacial polymerization kinetics and the density/accessibility of oxygen-containing groups within the XPS detection depth. Near-surface GO may also contribute to the oxygen signal.

**Figure 3:**
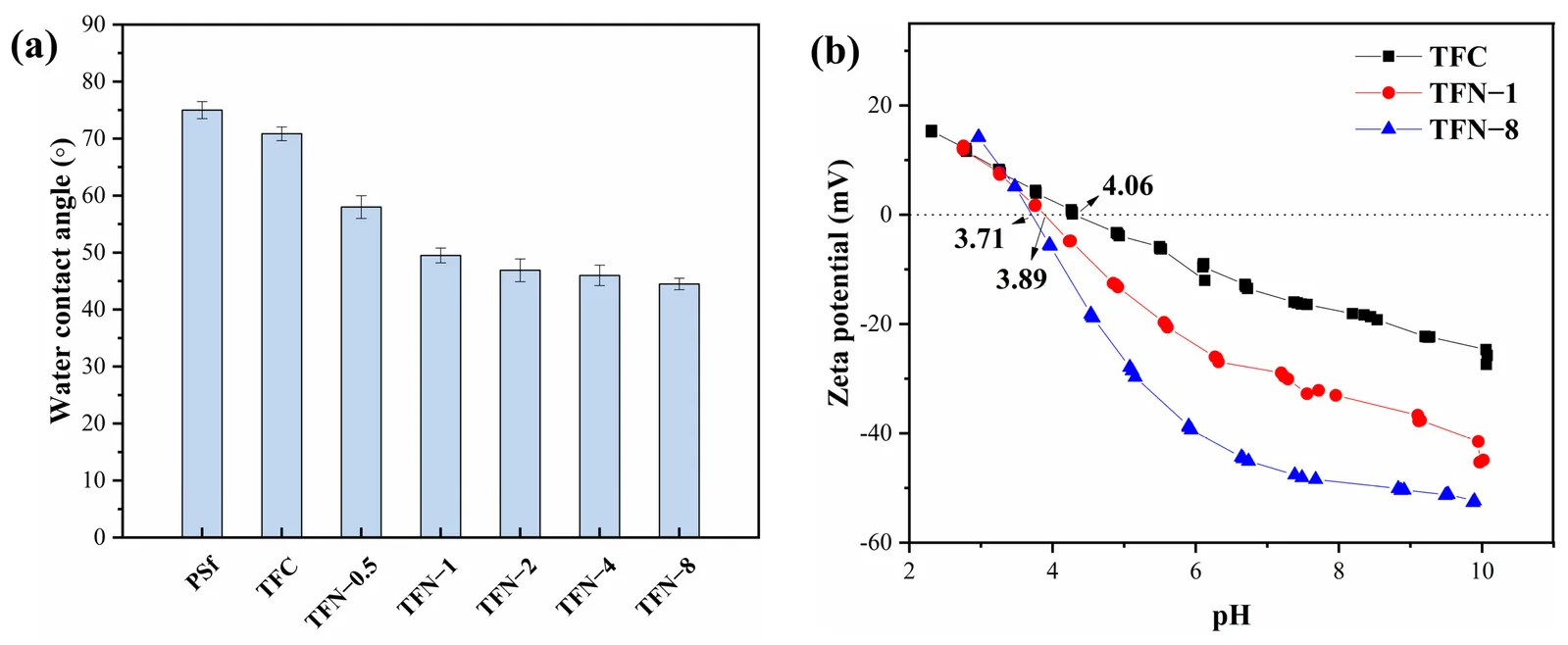
(**a**) Water contact angles of the membranes; (**b**) zeta potential of the TFC, TFN−1, and TFN−8 membranes.

Water contact angle measurements revealed a decrease from 70.9° on the TFC membrane to approximately 45° after GO incorporation (Figure 3a). This substantial improvement in hydrophilicity stems from the oxygen-rich polar groups on GO nanosheets, which facilitate extensive hydrogen bonding with water molecules, thereby rendering the surface more hydrophilic. While contact angle serves as a macroscopic indicator of surface affinity for water, its direct quantitative correlation with water sorption and permeability can be complex. We further calculated the water–membrane interaction energy to reflect the thermodynamic affinity of water with the membrane in Section 3.4. The surface charge properties of the membranes were characterized by zeta potential measurements (Figure 3b). The pristine TFC exhibited an isoelectric point (IEP) near pH 4.0. As GO loading rose, the IEP shifted toward lower pH values, reflecting greater surface electronegativity. This shift is associated with the introduction of carboxyl groups (-COOH) on the GO nanosheets, which deprotonate in aqueous solution, thereby enhancing the negative surface charge.

### 3.2. Membrane Morphology

**Figure 4:**
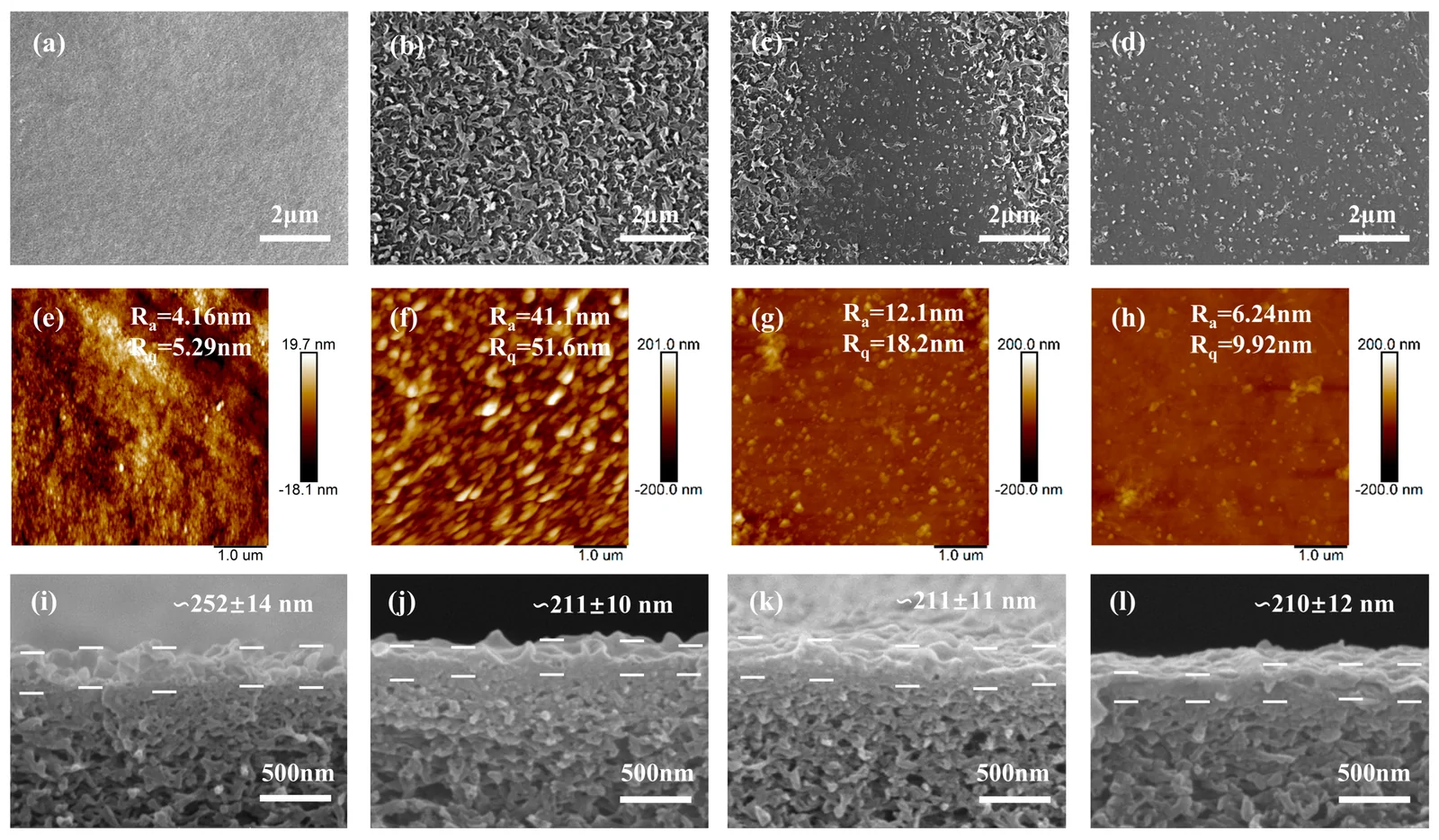
Surface SEM images of (**a**) PSf substrate; (**b**) TFC; (**c**) TFN−1; (**d**) TFN−8. AFM images of (**e**) PSf substrate; (**f**) TFC; (**g**) TFN−1 smooth domain; (**h**) TFN−8 smooth domain. Cross-sectional SEM images of (**i**) TFC; (**j**) TFN−1; (**k**) TFN−4; (**l**) TFN−8.

Top-view SEM and AFM images (Figure 4a–h) showed that the PSf substrate exhibited a smooth topography, with an arithmetic roughness (Ra) of 4.16 nm. The TFC membrane exhibited a typical leaf-like structure with an R_a_ of 41.1 nm, characteristic of rapid, exothermic interfacial polymerization. Upon incorporation of GO, the TFN membranes exhibited a heterogeneous surface comprising coexisting rough domains (traditional PA leaf-like structures) and smooth domains (GO-modulated regions). Figure S4 and Table S1 show the 3D images and surface roughness for the smooth domains in different TFN membranes. The smooth domains expanded progressively with increasing GO loading, coupled with a corresponding decrease in Ra from 12.1 nm to 6.24 nm. Cross-sectional SEM (Figure 4i,l) revealed a reduction in selective layer thickness from ~252 ± 14 nm in the pristine TFC membrane to ~211 ± 11 nm in TFN-1. This morphological transition is attributed to the kinetic modulation of IP by GO nanosheets, which act as diffusion barriers. In regions where GO nanosheets are densely packed or form a continuous interfacial layer, the MPD diffusion from the aqueous to the organic phase is significantly retarded by the strong hydrogen bonding with GO’s oxygen-rich groups. This diffusion retardation effect lowers the local reaction rate, suppressing the violent nucleation and growth processes that typically generate rough ridge-and-valley structures. Consequently, these GO-rich domains evolve into smoother and thinner PA layers. The progressive expansion of smooth domains with increasing GO loading further confirms this diffusion-retardation mechanism: as more GO nanosheets populate the interface, a greater fraction of the membrane surface experiences modulated IP kinetics.

### 3.3. PV Desalination Performance

**Figure 5:**
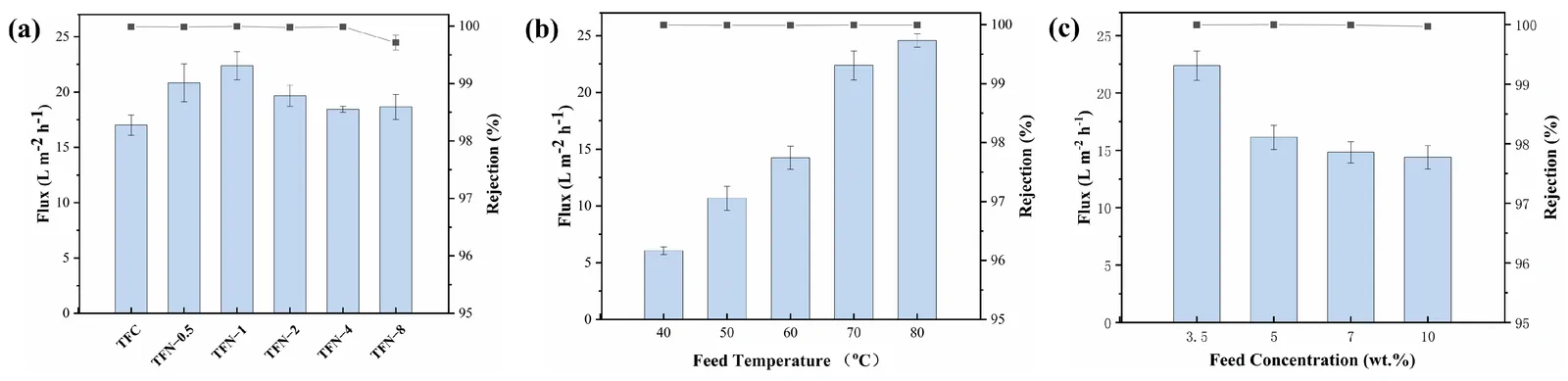
(**a**) PV desalination performance of the membranes: PV desalination performance of TFN−1 (**b**) at different feed temperatures and (**c**) at different feed concentrations.

Figure 5a shows that the water permeation flux rose from 17.02 L m^−2^ h^−1^ for the pristine TFC membrane to 22.38 L m^−2^ h^−1^ for the TFN-1 membrane, representing a 31% enhancement, without sacrificing NaCl rejection (>99.9%). The enhanced performance arises from the combined effects of improved membrane hydrophilicity, reduced selective layer thickness, and increased free volume, as investigated in the following section. However, increasing the GO concentration beyond 0.01 wt% caused a decline in water flux and a slight drop in rejection (to 99.75% for TFN-8). The expanding smooth surface at higher GO loadings may reduce the effective transport-active membrane area, thereby reducing permeability. The decrease in salt rejection is hypothesised to arise from GO aggregation at excessive loadings, which may disrupt the continuity of the PA matrix and introduce non-selective interfacial defects [42]. These defects might not be readily apparent in conventional surface SEM but are sufficient to affect the membrane’s separation efficiency. Future studies employing advanced characterization techniques will aim to provide more direct visualization of the distribution and aggregation behavior of GO within the PA selective layer. Based on these results, the optimal GO loading was determined to be 0.01 wt%. The TFN-1 membrane was further evaluated under varying operating conditions. The water flux increased from 13.5 to 24.6 L m^−2^ h^−1^ as the feed temperature rose from 50 to 80 °C, while salt rejection remained above 99.9% (Figure 5b). This trend is consistent with the increased water vapour pressure and enhanced polymer chain mobility at elevated temperatures. Increasing the feed NaCl concentration to 10 wt% reduced the flux from 22.38 to 14.4 L m^−2^ h^−1^ (Figure 5c). This decrease is attributed to reduced water activity at higher salt concentrations, which lowers the effective vapor-pressure driving force across the membrane. The salt rejection exceeded 99.9%, indicating that the GO-reinforced PA layer maintains its structural integrity under hypersaline conditions in short-term evaluations. As shown in Table 2, the TFN-1 membrane exhibits flux and rejection comparable to those of the recently reported GO-based membranes.

### 3.4. Solution-Diffusion Process from the Molecular Simulation Perspective

**Figure 6:**
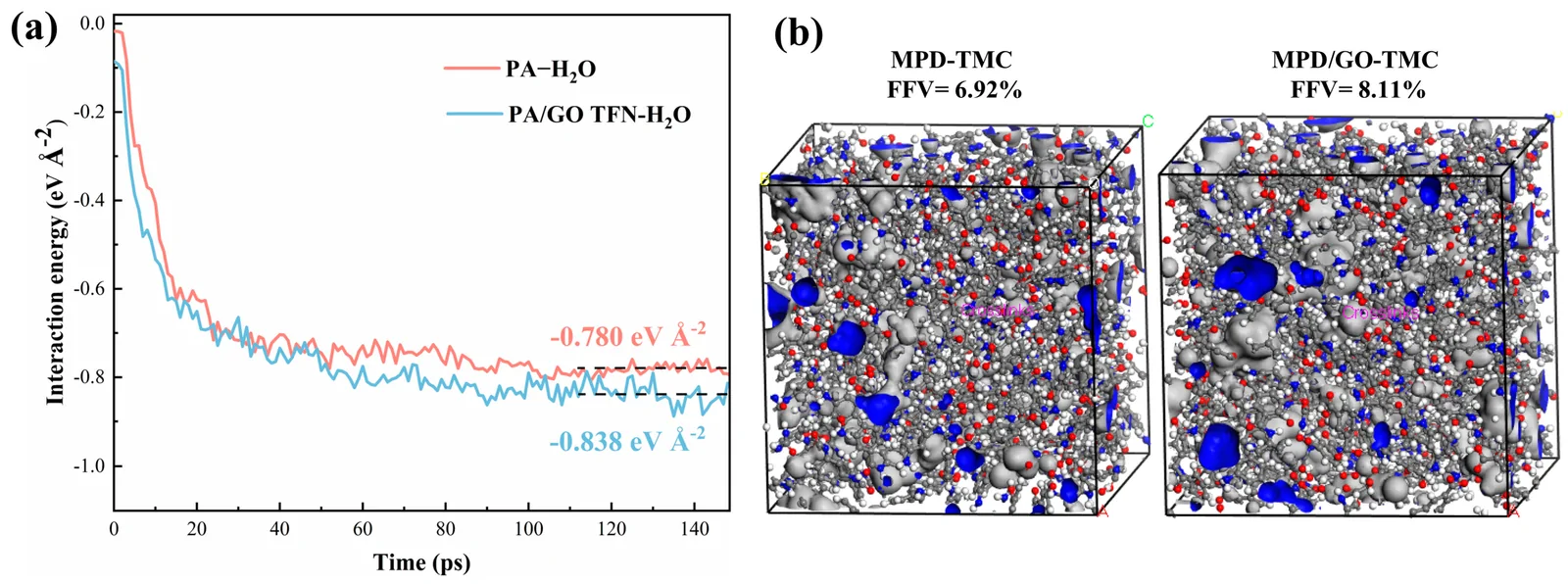
(**a**) Calculation of the water–membrane binding energy of PA TFC and PA/GO TFN membranes; (**b**) amorphous cells of the cross-linked PA and PA embedded with GO nanosheets.

In PV desalination, the mass transport of water through the membrane is generally described by a solution-diffusion model, in which the overall flux (J) is the product of the solubility coefficient (S) and the diffusivity coefficient (D) of the permeating water molecules within the membrane matrix. To investigate the molecular origins of the flux enhancement observed experimentally, MD simulations were performed to calculate the water–membrane interaction energy and the fractional free volume. The water–membrane interaction energy provides a thermodynamic indicator of water affinity, which correlates with the solubility coefficient, while the FFV serves as a structural proxy for available free space related to diffusivity [52]. As shown in Figure 6a, the water–membrane binding energy of the GO/PA composite membrane was significantly more negative (−0.838 eV Å^−2^) compared to that of the pristine PA membrane (−0.780 eV Å^−2^). The simulation also revealed a notable increase in the FFV of the GO/PA composite membrane (8.11%) compared to the pristine PA membrane (6.92%), as illustrated in Figure 6b.

The computational results showed that incorporating GO enhances the thermodynamic affinity between water molecules and the membrane matrix, facilitating water dissolution into the membrane. The stronger interaction can be attributed to the abundant oxygen-containing functional groups on GO nanosheets (hydroxyl, epoxy, and carboxyl groups), which form additional hydrogen bonds with water molecules. This finding corroborates the experimentally observed decrease in water contact angle, confirming that GO incorporation improves membrane hydrophilicity at the molecular level. In addition to the enhanced water affinity, the enlarged FFV in the GO/PA TFN membrane can be attributed to the disruption of compact PA chain packing by the rigid GO nanosheets, which creates additional interfacial voids and low-density regions within the polymer matrix. The enlarged FFV may provide more available space and potential pathways for water molecules to diffuse through the membrane, thereby reducing transport resistance. The molecular simulations supported the experimental increase in flux, though we note that this correlation is semi-quantitative and that absolute diffusion coefficients would require extended MD trajectories.

## 4. Conclusions

In conclusion, a GO/PA TFN membrane was prepared by incorporating GO nanosheets as aqueous additives during the IP process. The water flux of the optimized TFN-1 membrane exceeded that of the pristine TFC membrane by 31%, reaching 22.38 L m^−2^ h^−1^, with >99.9% NaCl rejection under standard test conditions (70 °C, 3.5 wt% NaCl). The TFN-1 membrane also sustained a flux of 14.4 L m^−2^ h^−1^ and rejection above 99.9% when the feed concentration reached 10 wt%. The observed performance enhancement can be attributed to the combined effects of improved surface hydrophilicity, reduced selective-layer thickness, and a modified membrane microstructure. Molecular dynamics simulations revealed that GO incorporation made the water–membrane binding energy more negative and increased the FFV, consistent with the enhanced water transport properties of the TFN membrane. It should be emphasized that the present results are based on short-term, laboratory-scale tests. Long-term continuous operation (≥24 h), cyclic regeneration, antifouling behavior, and potential GO leaching under realistic wastewater matrices have not been evaluated and remain essential future steps before the practical viability of these membranes can be assessed. Additionally, advanced characterization techniques could provide further insights into GO distribution, membrane stability, and potential structural changes after prolonged operation. Nevertheless, the facile fabrication strategy and the molecular insights provided by this work offer a useful reference for the continued development of TFN membranes in pervaporation desalination.

## Figures and Tables

**Table 1:** Composition of GO in MPD aqueous solutions for TFC and GO/PA TFN membrane preparation in this study.

Membranes	GO in Aqueous Solution (wt%)
TFC	0
TFN-0.5	0.005
TFN-1	0.01
TFN-2	0.02
TFN-4	0.04
TFN-8	0.08

**Table 2:** Summary of membrane performance with recently reported GO-based membranes.

Membranes	NaCl Conc. (wt%)	Expt. Temp (℃)	Thickness of Selective Layer	Flux (L m^−2^ h^−1^)	Salt Rej. (%)	Ref.
PA/GO/PAN	3.5	70	200–310 nm	26.7	99.99	[34]
PEI/GO LbL	2	65	-	13.7	>99.9	[35]
GO post-modified	3.5	79	~ 260 nm	22.6	99.99	[36]
PVA graft GO-PVA	3.5	30	1 μm	4.46	99.99	[43]
GOQDs-PVA	3.5	79.9	14.5 μm	17.09	>99.6	[44]
PVA/chitosan-coated GO composite	10	70	-	30.5	99.99	[26]
MB-GO	3.5	90	120 nm	27.2	>99.5	[45]
NH_2_-POSS@GO	3.5	80	204 nm	112.7	99.98	[46]
Geo-rGO-TiO_2_	3.5	60	-	2.92	91	[47]
MFGO	3.5	80	~ 5 μm	4.2	99.98	[48]
MFGO_GONR	3.5	80	~ 5 μm	6.8	99.98	[48]
MFGO_Full	3.5	80	~ 5 μm	4.9	99.98	[48]
E-CTA/EOGO	3.5	50	-	4.5	99.99	[15]
Chitosan/Ag NP/GO@BM	1	40	-	24.07	99.99	[49]
c-PSSA-GO	1	57	12.08 ± 0.52 µm	15.56	99.99	[50]
c-GOM-5/MCE	10	85	138 nm	91	52	[51]
GO/PA TFN-1	3.5	70	~ 210 nm	22.38	>99.9	This work

## Data Availability

The original contributions featured in this study are provided within the article and the Supplementary Materials. Additional inquiries can be addressed to the corresponding author.

## Supplementary Materials

The following supporting information can be downloaded at: https://www.mdpi.com/article/10.3390/membranes16100337/s1. Figure S1 [36]: (a) AFM and (b) SEM images of GO nanosheet obtained from the manufacturer; Figure S2 [41]: Chemical structure of (a) MPD; (b) TMC; (c) GO molecule; Figure S3: Snapshots of the membrane-water-vacuum system at 0 ps: (a) PA network; (b) PA embedded with 2 GO molecules; Figure S4: 3D images of the smooth domains in membranes from AFM: (a) TFC; (b) TFN-0.5; (c) TFN-1; (d) TFN-2; (e) TFN-4; (f) TFN-8; Table S1: Surface roughness of the smooth domain in membranes.

## Abbreviations

The following abbreviations are used in this manuscript:

AFM	Atomic force microscopy
ATR-FTIR	Attenuated total reflectance Fourier transform infrared
DI	Deionized
FFV	Fractional free volume
GO	Graphene oxide
IEP	Isoelectric point
IP	Interfacial polymerization
MD	Molecular dynamic
MPD	M-phenylenediamine
PA	Polyamide
PSf	Polysulfone
PV	Pervaporation
RO	Reverse osmosis
SEM	Scanning electron microscopy
TFC	Thin-film composite
TFN	Thin-film nanocomposite
TMC	Trimesoyl chloride
XPS	X-ray photoelectron spectroscopy
