# Supplementary material for: Graphene Oxide (GO)/Polyamide Thin-Film Nanocomposite Membranes for Enhanced Pervaporation Desalination: Role of GO in Microstructural Modification and Water Transport
Source: Membranes (Basel). 2026 Oct 11;16(10):337. doi: 10.3390/membranes16100337 (PMC13707347; doi:10.3390/membranes16100337)
Supplement: Supplementary file 1 [file membranes-16-00337-s001.zip › membranes-4558810-supplementary.pdf]

Supplementary Material

Graphene oxide (GO)/polyamide thin-film nanocomposite membranes for enhanced pervaporation desalination: Role of GO in microstructural modification and water transport

Yi Chen[1,*], Yu-xuan Li[2], Yaoli Guo[3], Xiaolong Xie[1], Pengwei Huang[1], Weini Chen[1], Shengchao Zhao[4,5,*]

1 Department of Chemical Engineering, Jieyang Polytechnic, Jieyang 522000, China
2 MayAir Technology (China) Co., Ltd., Nanjing 210000, China
3 State Key Laboratory of Radio Frequency Heterogeneous Integration, Shenzhen University, Shenzhen 518060, China
4 Institute for Advanced Study, Shenzhen University, Shenzhen, 518060, China
5 College of Civil and Transportation Engineering, Shenzhen University, Shenzhen, 518060, China
* Corresponding author.
E-mail address: 2016101054@email.szu.edu.cn , zhaoshch@szu.edu.cn

S1. Morphology of the commercial GO nanosheets

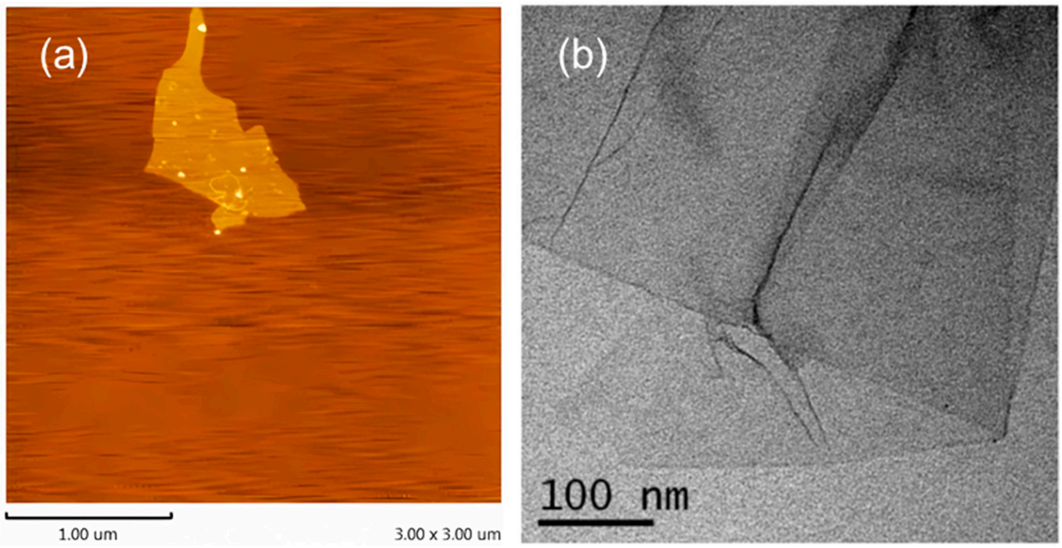

Figure S1. (a) AFM and (b) SEM images of GO nanosheet obtained from the manufacturer [1].

S2. Simulation details

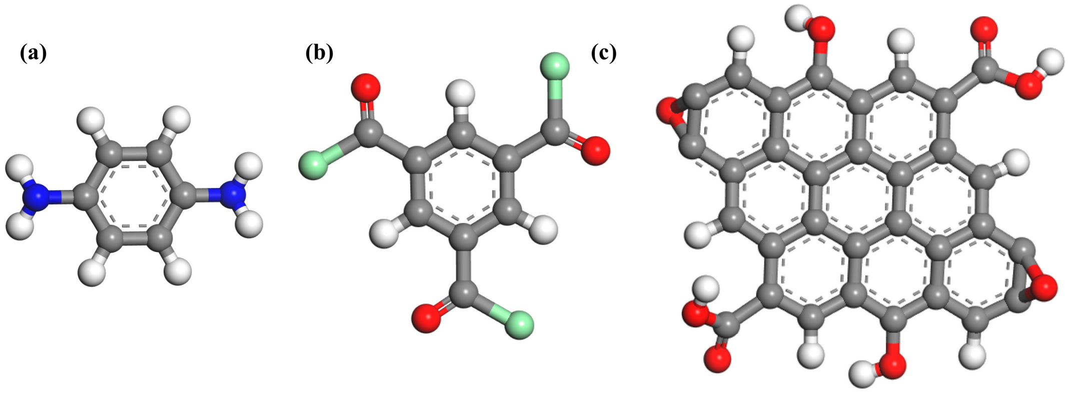

Figure S2. Chemical structure of (a) MPD; (b) TMC; (c) GO molecule

The PA network was constructed from 110 TMC and 165 MPD molecules at a molar ratio of 1:1.5 using the cross-linking script developed by Jason DeJoannis et al. The degree of cross-linking between the amino and acyl chloride groups was set to 70%. The resulting cross-linked amorphous cells then underwent a three-step kinetic relaxation [2]: (i) geometry optimization using the Forcite module; (ii) 100 ps of equilibration under the NVT ensemble; and (iii) 100 ps of simulation under the NPT ensemble to optimize the cell dimensions. To generate the GO/PA network, two GO models (molecular weight = 522.424) were inserted into the PA cross-linked cell, and the same relaxation protocol was applied.

S3. Calculation details

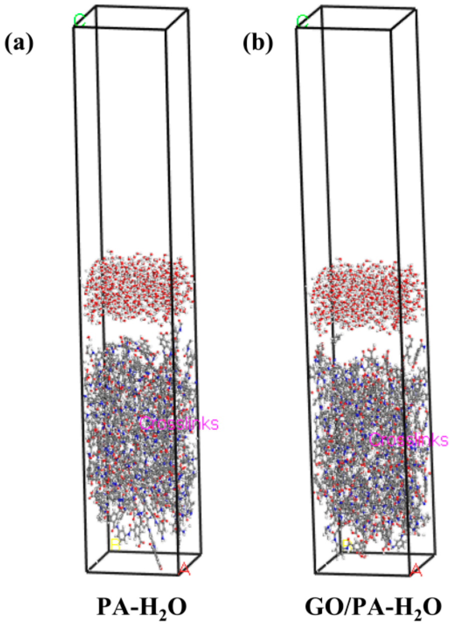

Figure S3. Snapshots of the membrane-water-vacuum system at 0 ps: (a) PA network; (b) PA embedded with 2 GO molecules

To determine the fractional free volume (FFV) of both the pure PA and GO/PA networks, the Connolly surface method was employed using a spherical probe 2.8 Å in diameter, corresponding to the geometric size of a water molecule. For the water - PA interaction energy simulations, an amorphous cell containing 530 water molecules was first constructed and then subjected to structural optimization via the same kinetic relaxation protocol used previously. A three-layer model was subsequently assembled, comprising (from bottom to top) a lower water layer (H_2O), a middle PA-based layer (either PA or PA/GO), and an upper vacuum layer 80 Å in thickness. After an additional 150 ps of kinetic equilibration under the NVT ensemble, the interlayer interaction energy between the PA-based layer and the water layer was computed using the script developed by Stephen Todd.

S4. Surface roughness and actual surface area of membranes from AFM

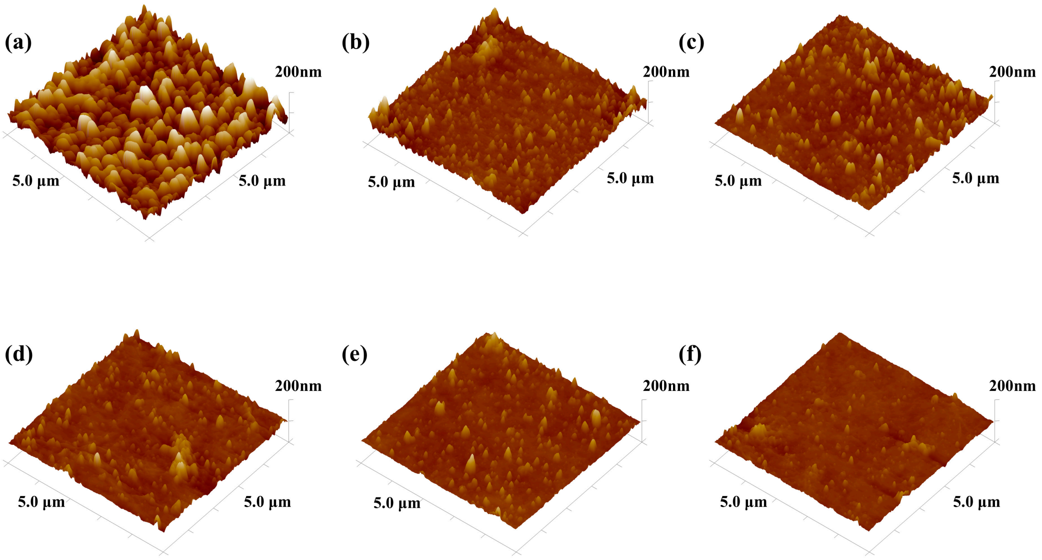

Figure S4. 3D images of the smooth domains in membranes from AFM: (a) TFC; (b) TFN-0.5; (c) TFN-1; (d) TFN-2; (e) TFN-4; (f) TFN-8

Table S1. Surface roughness of the smooth domain in membranes

Membrane	R_a (nm)	R_q (nm)
TFN-0.5	13.0	19.6
TFN-1	12.1	18.2
TFN-2	10.6	16.1
TFN-4	9.38	14.7
TFN-8	6.24	9.92

Reference

1. Zhao, S.; Sheng, J.; Chen, Y.; Wang, M.; Liu, Z.; Zhao, L.; Guo, Y.; Chen, K.; Yuan, B.; Li, P.; Niu, Q.J. Robust Polyamide-Based Composite Membrane with Controllable Graphene Oxide Post-Modification via Rod-Coating for Enhanced Pervaporation Desalination. *J. Environ. Chem. Eng.* **2025**, *13*, 115505, doi:10.1016/j.jece.2025.115505.

2. Zhao, S.; Peng, J.; Jin, C.; Zhao, S.; Chen, K.; Yuan, B.; Li, P.; Niu, Q.J. Robust Heterogeneous-Polyamide Based Reverse Osmosis Membrane Incorporated with Nonsolvent Induced Self-Assembled Dendrimer Aggregates. *Macromolecules* **2026**, *59*, 3188–3198, doi:10.1021/acs.macromol.5c03324.
